# Micro-Networks for Robust MR-Guided Low Count PET Imaging

**DOI:** 10.1109/TRPMS.2020.2986414

**Published:** 2020-04-08

**Authors:** Casper O. da Costa-Luis, Andrew J. Reader

**Affiliations:** Department of Biomedical EngineeringSchool of Biomedical Engineering and Imaging Sciences, St. Thomas’ HospitalKing’s College London 4616 LondonSE1 7EHU.K.

**Keywords:** Convolutional neural network (CNN), deep learning (DL), guided reconstruction, image processing, image reconstruction, machine learning, magnetic resonance (MR), maximum-likelihood expectation maximization (MLEM), resolution modeling (RM), resolution recovery, positron emission tomography (PET)

## Abstract

Noise suppression is particularly important in low count positron emission tomography (PET) imaging. Post-smoothing (PS) and regularization methods which aim to reduce noise also tend to reduce resolution and introduce bias. Alternatively, anatomical information from another modality such as magnetic resonance (MR) imaging can be used to improve image quality. Convolutional neural networks (CNNs) are particularly well suited to such joint image processing, but usually require large amounts of training data and have mostly been applied outside the field of medical imaging or focus on classification and segmentation, leaving PET image quality improvement relatively understudied. This article proposes the use of a relatively low-complexity CNN (micro-net) as a post-reconstruction MR-guided image processing step to reduce noise and reconstruction artefacts while also improving resolution in low count PET scans. The CNN is designed to be fully 3-D, robust to very limited amounts of training data, and to accept multiple inputs (including competitive denoising methods). Application of the proposed CNN on simulated low (30 M) count data (trained to produce standard (300 M) count reconstructions) results in a 36% lower normalized root mean squared error (NRMSE, calculated over ten realizations against the ground truth) compared to maximum-likelihood expectation maximization (MLEM) used in clinical practice. In contrast, a decrease of only 25% in NRMSE is obtained when an optimized (using knowledge of the ground truth) PS is performed. A 26% NRMSE decrease is obtained with both RM and optimized PS. Similar improvement is also observed for low count real patient datasets. Overfitting to training data is demonstrated to occur as the network size is increased. In an extreme case, a U-net (which produces better predictions for training data) is shown to completely fail on test data due to overfitting to this case of very limited training data. Meanwhile, the resultant images from the proposed CNN (which has low training data requirements) have lower noise, reduced ringing, and partial volume effects, as well as sharper edges and improved resolution compared to conventional MLEM.

## I. Introduction

Positron emission tomography (PET) image reconstruction is an ill-posed inverse problem, for which maximum likelihood expectation maximization (MLEM) is a commonly used iterative reconstruction method. Advantages of MLEM include the ability to incorporate a model of the entire acquisition process, including, e.g., attenuation and scatter.

Lowering the injected radioactive dose and/or overall scan time results in fewer acquired counts. Noise suppression becomes particularly important in the case of low count scans. As the sinogram data is inherently Poisson in distribution [1], both signal and variance are related to the total number of counts. Signal to noise ratio (SNR) thus is related to the root of the total number of counts [2]. Low count scans, therefore, result in images with high levels of noise.

Marked improvement in image detail (resolution and contrast recovery) and visual noise suppression can be achieved through use of resolution modeling (RM), apparently leading to better lesion detectability under certain conditions [3]–[6]. However, RM can also introduce ringing artefacts. The resultant visual impact on reconstructed images is extra edges parallel to those already in the image. These artefacts can greatly exaggerate maximum standardized uptake values (SUV_max_) which can lead to overestimation of tumor aggressiveness [7], [8]. There is, therefore, debate as to whether RM should even be used at all [5]. Under-modeling of resolution, post-smoothing (PS) [9], and regularization methods (such as total variation de-noising [10]) can compensate for reconstruction artefacts. However, these methods tend to degrade resolution or edge accuracy.

Alternatively, simultaneously acquired computed tomography (CT) or magnetic resonance (MR) data—which typically have lower noise—can be used in techniques such as nonlocal means (NLMs) to reduce noise in PET reconstructions [11]. Kernelized methods may even be incorporated into the MLEM reconstruction process [12].

This article proposes an alternative post-processing step informed by deep learning (DL)—specifically, deep convolutional neural networks (CNNs). CNNs are multilayer frameworks capable of learning high-level image features from pixel data. This builds on the concept of sparse representation of features used in dictionary learning approaches [13], [14]. CNNs are particularly suited to image processing tasks and have garnered much excitement in the computer science community. Meanwhile, CNNs applied to medical imaging have primarily focused on classification and segmentation [15], [16], and have left PET, in particular, relatively understudied [17]. Uptake of CNNs for medical imaging quality improvement has been comparatively recent and modest [18], [19], and typically applied to 2-D slices and/or patches [20]–[22]. Some proposals include combining DL with an unfiltered backprojection as a faster, comparable alternative to iterative MLEM reconstruction [23], while others suggest 2-D patch-based methods to reduce noise in low-dose PET-CT [24] and PET-MR reconstructions [25]. Recently, CNNs have also been incorporated into iterative reconstruction [26], [27]. CNN architectures are particularly well suited to using the increased resolution available in jointly acquired MR or CT data to reduce the noise in PET reconstructions. However, such networks typically require large amounts of training data and suffer from computational memory constraints.

For low dose PET-MR, small (5^3^) 3-D patches have been used in sparse dictionary-based approaches [28], [29]. For fully 3-D low dose PET-MR, non-CNN approaches such as regression forests have also been applied in prior work [30].

In contrast, this article focuses on improving image quality through 3-D CNNs which are flexibly designed to use MR guidance for reduced dose PET imaging, as well as remove reconstruction artefacts. Alternative methods may be used as additional network input channels, which should ensure superior performance. The primary aim is to reduce noise, while resolution improvement is secondary. Due to the design and resultant small size of the networks used here, we propose the term micro-network, or $\mu $-net. These $\mu $-nets have a comparatively small parameter space and thus are robust against overfitting on extremely limited training data sets, in stark contrast to the U-nets found in [31] and [32].

## II. Methods

The proposal is to use a neural network to improve the quality of low count reconstructions. Three cases are considered. Initially, a network is trained to map low count simulations to the ground truth. Second, the same network architecture is retrained to map to standard count reconstructions instead. Finally, this latter case is repeated with real patient data. The following section starts with a description of the simulated data.

### A. MLEM

#### 1) Simulations:

MR-based *BrainWeb* segmentations of 20 subjects [33] were modified to have [^18^F]FDG PET-like intensities (contrast ratio 4:1 for gray to white matter, 0.5:1 for dura, and ranging from 6:1 to 8:1 for spherical lesions of 5 to 15 mm in diameter and varying sharpness which were introduced into the phantom). The positions and sizes of these lesions were randomized [34]. Attenuation maps were generated with factors of 0.13 and 0.0975 for bone and tissue, respectively, and added to scanner manufacturer-provided hardware maps. Some randomized structure was also introduced for the PET and MR segmentations according to (1) to produce a realistic non piece-wise constant phantom $\boldsymbol {\tau }$, given by

\begin{equation*} \boldsymbol {\tau } = \boldsymbol {\phi } \circ \left ({\boldsymbol {1} + \gamma \left [{2G_{\sigma }\left ({\boldsymbol {\rho }}\right ) - \boldsymbol {1}}\right ]}\right )\tag{1}\end{equation*}

**where**
Abbreviation: Expansion
$\boldsymbol {\tau }$: is used as a realistic ground truth phantom for the simulations;
$\boldsymbol {\phi }$: is a *BrainWeb*-based segmented phantom;
$\gamma $: is an intensity parameter chosen to be 1.5 for PET and 1 for MR segmentations;
$G_{\sigma }$: represents Gaussian smoothing of $\sigma =\mathrm {1~pixel}$;
$\boldsymbol {\rho }$: is of the same size as $\boldsymbol {\phi }$ with random uniform distributed elements $\in [0, 1)$;
$\circ $: is the Hadamard (element-wise) product.

For each phantom, resolution degradation effects were simulated in image space by smoothing with a Gaussian with 4.5-mm full width at half maximum (FWHM). A forward projector from *NiftyPET* [35] was then used to simulate 837 span 11 sinograms $\boldsymbol {m}$. Simulations correspond to the Siemens Biograph mMR scanner ($2.09\times 2.09\times $ 2.03 mm^3^ voxel size and image dimensions $344\times 344\times 127$), accounting for photon attenuation and normalization (including geometry, crystal efficiencies, and dead time effects as described in [35]).

Count levels were varied from 3 M up to a maximum of 300 M (including 26 % randoms and 28 % scatter). The maximum count level was chosen to be comparable to that of real data (for a scan of 20 min with 370 MBq injected activity). Ten Poisson noise realizations were generated for each noise level, followed by MLEM reconstructions. Each iteration $k$ of the reconstructed image $\boldsymbol {\theta }$ is given by

\begin{equation*} \boldsymbol {\theta }^{\left ({k+1}\right )} = {\frac{\boldsymbol {\theta }^{\left ({k}\right )} }{ \boldsymbol {H}^{T}\boldsymbol {X}^{T}\boldsymbol {1}}} \circ \boldsymbol {H}^{T}\boldsymbol {X}^{T} {\frac{\boldsymbol {m} }{ \boldsymbol {XH}\boldsymbol {\theta }^{\left ({k}\right )}+\boldsymbol {\varrho }}}\tag{2}\end{equation*}

**where**
Abbreviation: Expansion
$\boldsymbol {\theta }^{(k)}$: is the reconstructed image at the $k^{\mathrm{ th}}$ iteration;
$\boldsymbol {H}$: can be used to include an RM kernel;
$\boldsymbol {X}$: is the rest of the system matrix (forward projection including attenuation and normalization);
$\boldsymbol {m}$: is the sinogram data;
$\boldsymbol {\varrho }$: represent randoms and scatter, and division is Hadamard (performed element-wise).

For all data sets, 300 MLEM iterations were performed with RM, and 100 iterations without RM [$\boldsymbol {H}=\boldsymbol {I}$ in (2)]. More iterations are required for RM due to its lower rate of convergence. RM reconstructions use a Gaussian point spread function (PSF) of 4.5 mm FWHM in image space. Corresponding MR data was obtained by adding randomized structure (1) to the T1 *BrainWeb* phantoms and downsampling to the same resolution and dimensions as the PET reconstructions. The randomized structure ensures that a simple mapping from T1 to ground truth PET is not possible.

As a reference method, reconstruction results are post-smoothed with a Gaussian kernel. It should be noted that smoothing using a kernel at least as large as the PSF has long been proposed as a way of obviating ringing artefacts [9], [36].

A further reference is provided by modifying the NLMs algorithm [37] to perform MR-guided Gaussian-weighted filtering using the T1-weighted reconstruction $\boldsymbol {\theta }^{(\text {T1})}$. The NLM output is defined to be

\begin{align*} \mathrm {NLM}\left ({\theta ^{\left ({k}\right )}_{j}}\right )=&{\frac{\sum _{i\in N_{j}} w_{i,j} \theta ^{\left ({k}\right )}_{j} }{ \sum _{i\in N_{j}} w_{i,j}}} \tag{3}\\ w_{i,j}=&\exp \left \{{-{\frac{1}{ 2}}\left ({ {\frac{\theta ^{\left ({\text {T1}}\right )}_{i}-\theta ^{\left ({\text {T1}}\right )}_{j}}{ \Omega }}}\right )^{2}}\right \}\tag{4}\end{align*}

**where**
Abbreviation: Expansion
$\theta ^{(k)}_{j}$: is the $j^{\mathrm{ th}}$ voxel of an MLEM PET reconstruction from (2);
$w_{i,j}$: is a T1-derived weighting factor;
$N_{j}$: is the $5\times 5\times 5$ neighborhood around $j$;
$\theta ^{(\text {T1})}_{i}$: is the $i^{\mathrm{ th}}$ voxel of the T1-weighted MR reconstruction;
$\Omega $: is an optimization parameter.

Ten noise realizations and reconstructions are performed for all phantoms to enable calculation of standard deviation values $\sigma $ across realizations. Bias $b$ and normalized root mean squared error (NRMSE) $\epsilon $ are also calculated. These metrics are all normalized as in [38]. Normalization is done in a manner which avoids element-wise division (thereby avoiding exaggeration from low intensity values) and to be consistent with $\epsilon ^{2} = \sigma ^{2} + b^{2}$

\begin{align*} b=&{\frac{100\%}{ \sqrt {\sum _{j} T_{j}^{2}}}}\sqrt {\sum _{j} \left ({ T_{j} - \underset {r}{\mathrm {E}}\left \{{ \theta _{r,j} }\right \} }\right )^{2}}\tag{5}\\ \sigma=&{\frac{100\%}{ \sqrt {\sum _{j} T_{j}^{2}}}}\sqrt {\sum _{j} \underset {r}{\mathrm {Var}}\left \{{ \theta _{r,j} }\right \}}\tag{6}\end{align*}

and

\begin{equation*} \epsilon = {\frac{100\%}{ \sqrt {\sum _{j} T_{j}^{2}}}}\sqrt { \sum _{j} \underset {r}{\mathrm {E}}\left \{{ \left ({T_{j} - \theta _{r,j}}\right )^{2} }\right \}}\tag{7}\end{equation*}

**where**
Abbreviation: Expansion
$\theta _{r,j}$: is the $j^{\mathrm{ th}}$ voxel of the $r$th reconstruction (from the $r^{\mathrm{ th}}$ noise realization);
$ \mathop{\mathrm {E}}\limits_{r}\left \{{ \cdot }\right \}$: is the mean operator across $r$;
$ \mathop{\mathrm {Var}}\limits_{r}\left \{{ \cdot }\right \}$: is the variance operator across $r$;
$T_{j}$: is the $j^{\mathrm{ th}}$ target voxel;
$b$: is normalized bias;
$\sigma $: is normalized standard deviation;
$\epsilon $: is normalized root mean squared error (NRMSE).

#### 2) Real Data:

Real data $\boldsymbol {m}$ was also obtained from 10 [^18^F]FDG PET head scans using the same scanner. Count levels varied from 400 M to 500 M. Using *NiftyPET*, listmode data is randomly sampled with replacement (bootstrap method from [39]) at 300 M (standard), 30 M (low), and 3 M (very low) counts for each patient to ensure consistent count levels and similar distributions. Randoms were estimated through variance reduction of delayed coincidences [40], while scatters were updated (using a single-scatter model) at each MLEM iteration. On average, it was estimated that 28 % of the counts were scatter and 26 % were randoms. Each count level is sampled ten times for estimation of standard deviation for comparison purposes. Reconstructions were performed using the same method as with simulations [MLEM as per (2)]. The original raw listmode data (without bootstrap sampling) was also reconstructed for each patient in lieu of a known ground truth reference.

Corresponding MPRAGE T1 reconstructions were scaled and registered to the full count PET reconstructions using *dipy* [41] before performing NLM filtering on the PET reconstructions (3).

### B. Deep Convolutional Neural Networks

In this section, the low count PET reconstructions are combined with the corresponding MR reconstruction to form the network training input $\boldsymbol {\alpha }^{(1)}$ in (8) below. The network parameters are then optimized to minimize the difference between the current output [for layer $j=4$, this is $\boldsymbol {\alpha }^{(4)}$] and the desired target $\boldsymbol {T}$. This target may be either the ground truth $\boldsymbol \tau $ (if available) or standard (300 M) count reconstruction $\boldsymbol {\theta }^{(100)}_{\text {std}}$. For comparison, the Gaussian PS FWHM and the NLM parameter $\Omega $ are also both optimized on the same data.

#### 1) Layers:

Each layer $j$ of the network transforms its input vector $\boldsymbol {\alpha }^{(j)}$ as shown in

\begin{equation*} \boldsymbol {\alpha }_{k,r}^{\left ({j+1}\right )} = A_{j}\left ({ \beta _{k}^{\left ({j}\right )}\boldsymbol {1} + \sum _{i=1}^{n_{j-1}} \boldsymbol {\kappa }_{i,k}^{\left ({j}\right )} \boldsymbol {\alpha }_{i,r}^{\left ({j}\right )} }\right ) \quad \forall \,k\in \left [{1, n_{j}}\right ]\tag{8}\end{equation*}

**where**
Abbreviation: Expansion
$\boldsymbol {\alpha }_{i,r}^{(j)}$: is the $i^{\mathrm{ th}}$ channel of the $r^{\mathrm{ th}}$ low count noise realization input for layer $j$; such that $\boldsymbol {\alpha }^{(1)}$ represents the network’s inital input volumes;
$\boldsymbol {\kappa }_{i,k}^{(j)}$: is a matrix applying the $k^{\mathrm{ th}}$ kernel’s convolutional weights;
$n_{j}$: is the number of kernels (and therefore output channels);
$\beta _{k}^{(j)}$: is a bias (offset);
$A_{j}$: is a nonlinear element-wise activation function, here chosen to be sigmoidal: $A_{j}(x) = 1 / (1 + e^{-x})$, except for the last layer, where: $A_{3}(x) = \{x{~\text {for }}x>0,{~\text {and }}e^{x}-1{~\text {otherwise}}\}$.

$\boldsymbol {\alpha }^{(j)}$ represents a multichannel set of volumes. In the case of the network’s input, $\boldsymbol {\alpha }^{(1)}$, each channel could be a reconstructed modality volume, such as low count $\boldsymbol {\theta }^{(100)}$ or $\boldsymbol {\theta }^{(\text {T1})}$.

For a given layer $j$, we will use $n_{j}$ to denote the number of kernels and $s_{j}$ to denote width of each kernel. The number of output channels of a layer is given by the number of kernels, and is thus also $n_{j}$.

$\boldsymbol {\kappa }^{(j)}$ corresponds to $n_{j}$ different multichannel kernels (each with $n_{j - 1}\times s_{j}\times s_{j}\times s_{j}$ parameters) each operating on the $n_{j-1}$-channel input $\boldsymbol {\alpha }^{(j)}$ to produce a corresponding output channel in $\boldsymbol {\alpha }^{(j+1)}$. Each output channel can be considered to be a feature map, with the sensitivity of the corresponding feature-detecting kernel controlled by the combination of $\boldsymbol {\beta }^{(j)}$ and $A_{j}$ (nonlinear thresholding). $A_{j}$ is often chosen to be rectified linear units (ReLU)—setting negative values to zero—which performs computationally fast thresholding by simply discarding data. However, in the micro-network proposed here, such discarding is not desirable as it would result in minimal computational speed improvements at the cost of accuracy. Using sigmoids ensures that information is retained as it propagates through the network, and is discussed in more detail in Section II-C. The final layer utilizes an exponential linear unit (ELU [42]) as a desirable exclusively lower-bound constraint. This acts as a weak non-negativity constraint without introducing nonlinearities for positive values.

#### 2) Micro-Net:

The multichannel input $\boldsymbol {\alpha }^{(1)}$ used here includes $\boldsymbol {\theta }^{(\text {T1})}$ as well as two independent low count PET reconstructions $\boldsymbol {\theta }^{(100)}$ and $\boldsymbol {\theta }^{(300)}_{\text {RM}}$ of the same single noisy dataset. This presents the network with additional useful information—lower noise RM images as well as RM-artefact-free standard MLEM. Post-smoothed versions were not provided as the convolutional network itself is trivially capable of performing optimal (to an extent determined by the training process) spatially invariant smoothing. T1-guided NLM was applied to the RM PET reconstruction $\boldsymbol {\theta }^{(300)}_{\text {RM}}$ using (3) and also provided as an input. This allows for modulation of the PET data by the MR intensities, thereby sharing edge information. Closely approximating such an operation would normally require, e.g, greater network density (increasing $s_{j}$). However, this would unnecessarily greatly increase the number of optimization parameters, thus increasing computational cost and the likelihood of overfitting on limited training data sets. Alternatively, a sufficiently deep network (increasing $n_{j}$) could also achieve the overall effect of every input pixel potentially affecting every output pixel. Adding such depth would, however, have the same caveat (as increasing $s_{j}$) of having many optimization parameters.

In total, there are four different input volumes [subscripted by $i$ in $\boldsymbol {\alpha }_{i,r}^{(1)}$ from (8)]: $\boldsymbol {\theta }^{(100)}$, $\boldsymbol {\theta }^{(300)}_{\text {RM}}$, $\boldsymbol {\theta }^{(\text {T1})}$, and $\mathrm {NLM}(\boldsymbol {\theta }^{(300)}_{\text {RM}})$, each of which are independently normalized (offset to have zero mean and scaled to have unit variance). The exception is the last case, where only the input to the NLM filter is normalized. The target is normalized to have unit variance (but no alteration of mean). This justifies the final layer’s ELU activation function: large negative values should not be expected, and there should be no upper bound. This is discussed in more detail in Section II-C1. Normalization allows the network to benefit from both PET and MR information despite their large intensity distribution differences [43].

**Fig. 1.**
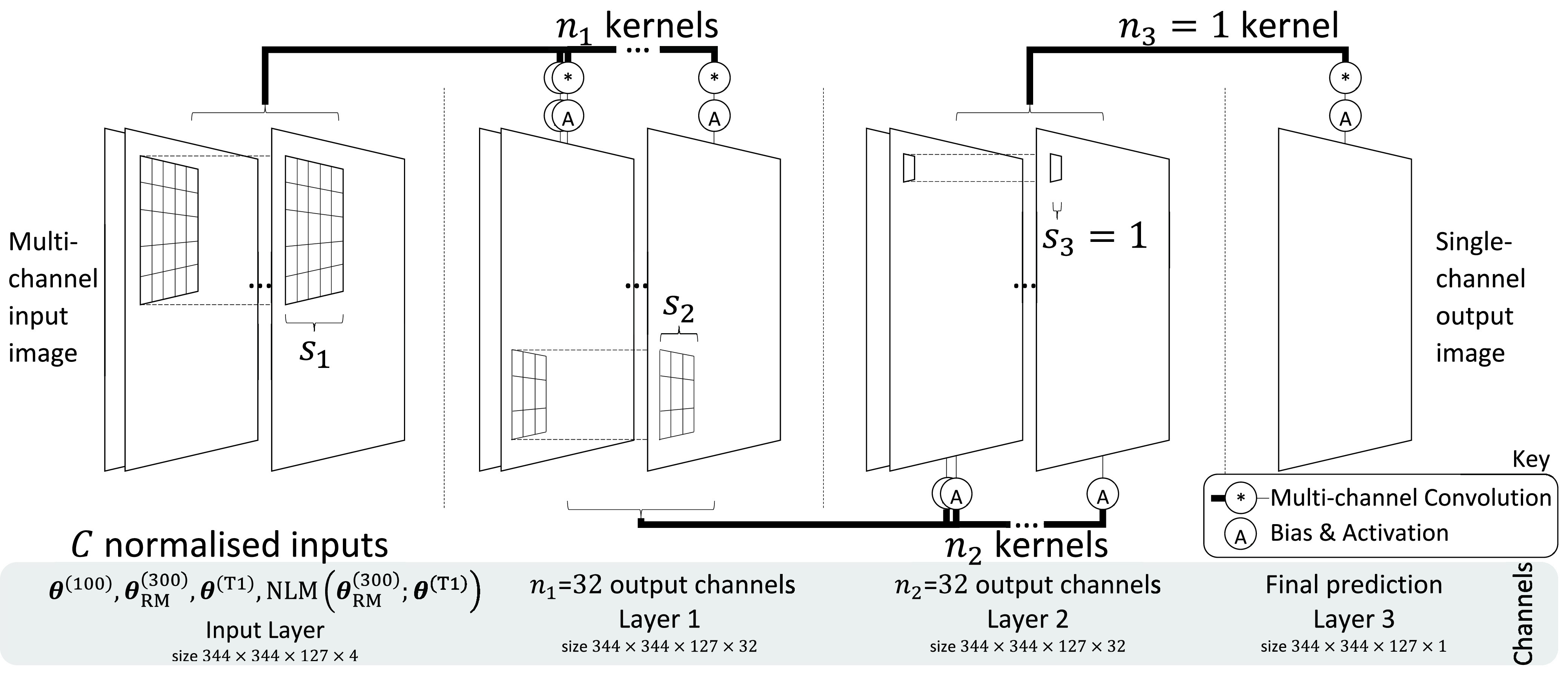
Visualization of 3-layer $\mu$-net architecture. Note that 3-D volumetric channels are depicted as 2-D to ease understanding. “multichannel convolution” is a many-to-one-channel operation identical to the element-wise sum of each input channel convolved with its own unique kernel. There are $n_{j}$ unique kernels in each layer $j$. Convolutions are performed with stride 1 and zero padding on whole volumes without patching. For $n=\{32, 32, 1\}$ and $s=\{5, 3, 1\}$ applied to $C=4$ input volumes, there are 43 745 parameters in total.

The main network proposed here consists of three layers, with $n_{1}=32, n_{2}=32$, and $n_{3}=1$, while $s_{1}=5, s_{2}=3$, and $s_{3}=1$. The workflow to post-process with a pretrained network would be first to normalize inputs, obtain a network prediction, and then multiply by a constant such that the total intensity matches the prenormalized input. A visualization of the network architecture is shown in Fig. 1.

For comparison, different networks were trained for various choices of $n_{1}$ and $n_{2}$. The rationale is that the first layer performs detection of up to $n_{1}$ different features, and the second recombines these feature maps in different ways to produce $n_{2}$ candidate PET volumes. The final layer performs a weighted average over these volumes. The network, therefore, has comparatively few parameters [$\mathcal {O}(10^{4})$]. As the number of parameters is much lower than the size of the training data [which is $\mathcal {O}(10^{7})$ even if compressed], there is no risk of overfitting, since the network is incapable of memorizing the training data. This helps ensure that the network only performs feature recognition, as desired, rather than object generation. Ideally, if simulated features accurately represented real data, this would allow for training on simulated data and clinical application on real patient scans.

We propose the term micro-network or $\mu $-net to refer to such networks which are created to be small and robust to minimal amounts of training data by design. Adding more layers to increase complexity and network depth can rapidly increase test error. Such degradation can be due to increased optimization difficulty, and not necessarily due to overfitting [44]. Relatively shallow autoencoders perform better than deep U-nets, particularly when training data is limited [31].

Initially, two low count noise realizations of the same phantom or patient were used to create $R=2$ sets of reconstructions [subscripted by $r$ in $\boldsymbol {\alpha }_{i,r}^{(1)}$ from (8)]. The network is trained by minimizing the difference between the desired target $\boldsymbol {T}$ and the current output $\boldsymbol {\alpha }^{(4)}$. This is done in batch mode (simultaneously for both sets of reconstructions). A third reconstruction set from a different phantom or patient was also used to evaluate a validation value of the loss. Training is terminated when this validation value fails to decrease for 10 k epochs. The network state corresponding to minimum validation loss (10 k epochs before termination) is then restored. The training process involves the estimation of parameters $\boldsymbol {\kappa }$ and $\boldsymbol {\beta }$ by the iterative minimization—via gradient descent^1^—of the objective function (loss) $L$ (9), proportional (up to a constant) to the sample NRMSE (7), our chosen metric of interest in this article. The loss is given by

\begin{equation*} L\left ({\boldsymbol {\kappa }, \boldsymbol {\beta }; \boldsymbol {\alpha }^{\left ({1}\right )}, \boldsymbol {T}}\right ) = \sqrt {\frac{1}{ R\sum _{r=1}^{R}\left \|{\boldsymbol {T}_{r} - \mu _{\boldsymbol {\kappa },\boldsymbol {\beta }}\left ({\boldsymbol {\alpha }_{\cdot ,r}^{\left ({1}\right )}}\right )}\right \|^{2}}}\tag{9}\end{equation*}

**where**
Abbreviation: Expansion
$\boldsymbol {\alpha }_{\cdot ,r}^{(1)}$: is the input set of 4 volumes for the $r$th (low count) PET noise realization;
$\mu _{\boldsymbol {\kappa }, \boldsymbol {\beta }}$: represents the application of the micro-net, such that $\mu _{\boldsymbol {\kappa }, \boldsymbol {\beta }}(\boldsymbol {\alpha }_{\cdot ,r}^{(1)}) = \boldsymbol {\alpha }_{1,r}^{(4)}$;
$R$: is the total number of low count noise realizations;
$\boldsymbol {T}_{r}$: is the target PET output.

Trained using Tensorflow v2.0.0 [45] on an NVIDIA Quadro P6000, using the adaptive moment estimation (Adam) optimizer [46] with a learning rate of 10^−3^.

At the start of training, the weights and biases ($\boldsymbol {\kappa }, \boldsymbol {\beta }$) must be assigned starting values. *He* normal initialization [47] was used as it was found to reduce loss by a factor of 3 compared to *LeCun* uniform initialization [48]. The former method entails initializing $\boldsymbol {\kappa }^{(j)}$ by random normal sampling with standard deviation $\sqrt {2 / n_{j-1}}$, while biases $\boldsymbol {\beta }^{(j)}$ are set to zero. This helps prevent saturation of activation functions with very large positive or negative values.

The network’s biases make it possible to trivially correct for spatially invariant bias in the input PET images. Spatially invariant variance due to noise, however, should be accounted for by other aspects of the network’s design, so we believe a loss function susceptible to noise (in contrast to $\ell _{1}$) is acceptable. Specifically, robustness to spatially invariant noise is achieved by having a small architecture: the network here is certainly not dense; instead consisting of small local kernels which must be spatially invariant as they are applied to the whole input. As the kernels are optimized over the entire input, they must be able to cope with the various instances of noise found over the whole volume. The training phase should result in kernels optimized for the “average” region, which by definition has zero variance due to noise. Kernels should thus be able to compensate for spatially invariant noise irrespective of the chosen loss function.

Since the CNN has a small receptive field (small neighborhood width of seven input voxels which could affect an output voxel) applied over a large volume (two orders of magnitude wider than the receptive field) it seems logical that they should not be able to compensate for spatially variant noise. However, it is possible that based on the features detected in different spatial regions, kernels may indeed be activated by (and thus “aware of”) different spatial regions, thereby handling both spatially variant noise and bias.

While the primary objective here is to post-compensate for noise degradation, the CNN can also suppress artefacts, including the partial volume effect (PVE) and ringing.

#### 3) U-Net:

For comparison, a U-net is modified to have some of the advantages of the proposed $\mu $-net (Section II-C). These advantages include accepting normalized multichannel inputs, as well as performing fully 3-D convolutions. Optimization details (choice of optimizer, parameter initialization, and NRMSE loss) are kept the same as for the micro-net.

Specifically, the U-net comprises of an “encoder” and “decoder,” and a final residual layer. The encoder consists of four convolutional layers (with stride 2). The decoder repetitively performs trilinear upsampling (scale factor 2), concatenation with the corresponding encoder layer, and convolution (stride 1). The number of kernels per convolution layer is increased with U-net depth: $n=\{32, 64, 128, 256, 128, 64, 32, 1\}$. ELU activation functions are inserted for each multichannel convolution output.

The final residual layer adds the decoder’s single-channel ($n_{8}=1$) output (element-wise) to the NLM input channel (as this is the “best” input in terms of NRMSE).

### C. Contributions

This article builds on and provides a novel combination of methods found in the current literature.

#### 1) Activation Functions:

We use sigmoidal activation functions $A_{j}$ (8) that introduce nonlinear kernel sensitivity control. Compared to the more widely used ReLU (which sets negative values to zero), this is accomplished without discarding information. Note that the network’s inputs and targets are normalized and thus sigmoids (which have upper bounds unlike ReLU) should not introduce quantification errors. Sigmoids are also easier to optimize using backpropagation due to finite curvature and a nonzero gradient, and achieve similar benefits to batch normalization [43], [49] such as enabling higher learning rates and acting as a regularizer, thereby reducing the chance of overfitting and removing the need for dropout.

The benefit of using sigmoids (particularly for $\mu $-nets) outweighs the increased training time compared to ReLU. Furthermore, sigmoids also saturate gradually (unlike ReLU) and thus reduce the likelihood of “deactivation” (feature maps being set to zero regardless of the input data). With the relatively small architecture proposed here, there is a low amount of redundancy built into the network, and thus such deactivation should be less encouraged.

It should however be noted that the target output (whether the ground truth or MLEM reconstruction) is strictly positive. The final layer thus requires a different activation function. However, using an ReLU in the final layer (while it may enforce this consistency) is not advisable. Optimization becomes very difficult due to the sparse or “dying” ReLU problem [50], [51]. An ELU activation function is used instead. This introduces a softer minimum threshold for negative values (−1 rather than 0), while remaining linear for positive values. Compared to ReLU variants (including leaky, parametric, and randomized leaky ReLU), ELU has been shown to be more robust to noise and easier to optimize [42].

We found that enforcing strict non-negativity—by adding an offset of 1 or by using a plain exponential function in lieu of ELU—encourages undesirable saturation of the sigmoids in previous layers.

#### 2) Fully 3-D:

Using 3-D volumes (rather than 2-D slices) means adjacent slice information is available to kernels, resulting in a superior ability to correct PVEs and distinguish between signal and noise.

#### 3) Multiple Realizations:

For a given input noise level, training on more than one noise realization of the same patient ($R>1$) further increases robustness to noise at the chosen level, and reduces the need for more training data. This helps the network to detect variance and remove noise. The effect of using fewer ($R=1$) or more ($R=3$) training realizations is also investigated, with the expectation being that more realizations will increase network performance.

#### 4) No Patches or Downsampling:

Working directly on the full volumes (without subdivision into small regions and not pooling) ensures that all available data is used, without ignoring boundaries of small patches (which reduces use of adjacent voxel information to compensate for noise and PVE) and without downsampling (losing resolution unless skip connections are present). Additionally, zero padding is safe to use for convolutions without introducing edge artefacts as the whole volume is naturally zero at all boundaries. In contrast, using patches would require careful handling of edges.

#### 5) Unity Strides and No Augmentation:

Convolving with unity stride helps remove the need for data augmentation. Augmentations such as mirroring and rotating—which do not genuinely provide more information—also encourage rotational invariance even when the underlying system and features are not necessarily rotationally symmetric.

#### 6) Competitive Inputs:

A framework which allows for alternative methods (such as NLM) to be input channels theoretically guarantees superior performance (subject to appropriate learning rates and sufficient training data). This allows the network to act as a further refinement on preprocessed input channels. It also reduces the need for density and depth. For example, NLM allows for joint edge modulation across modalities—but this would require an element-wise product between input channels—which is something a CNN can only approximate if sufficiently dense and deep. To avoid this unnecessary increase in parameters to optimize, these competitive methods may be precomputed and supplied as inputs.

#### 7) Optimal Depth and Density:

The effects of varying the total number of layers, and varying the number of kernels per layer are investigated; and a network architecture is selected accordingly. It is found that a comparatively low number of kernels $n_{j}$ is sufficient in each layer. This avoids redundant parameters and precludes the possibility of overfitting (memorizing the training data rather than learning features). The number of optimization parameters in a layer $j$ is given by $(n_{j-1} \times s_{j}^{3} + 1)\times n_{j}$, meaning there are a comparatively small number of parameters (43 745) in total. The training data size is $\mathcal {O}(10^{7})$ even when compressed; which is impossible for the network to memorize.

## III. Results

The proposed and rival methods were first optimized on simulation data subjects for various count levels and targets. For testing, low count datasets from other subjects (not used during the training stage) were given to the network to make predictions for comparison to competitive methods. This process was then repeated for real data.

### A. Simulations

**Fig. 2.**
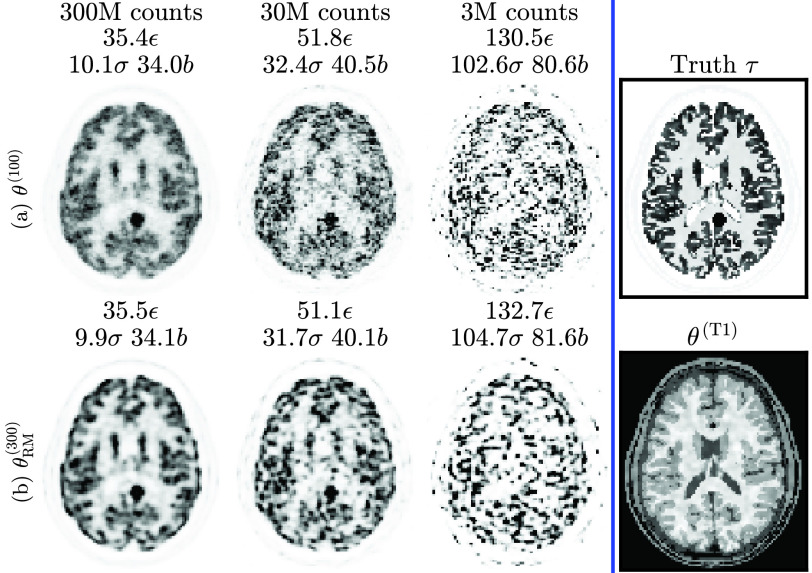
Simulation training data: cropped central slices from one set of MLEM reconstructions of subject 4 at different count levels. Left panel: row a) 100 iterations of MLEM, $\boldsymbol{\theta}^{(100)}$ (showing high noise), row b) 300 iterations with RM, $\boldsymbol{\theta}^{(300)}_{\text{RM}}$ (showing ringing, particularly in the gray matter at the cortical edge). NRMSE [$\epsilon$, (7)] and bias [$b$, (5)] are calculated versus the ground truth ($\boldsymbol\tau$, right panel). Standard deviation [$\sigma$, (6)] is calculated across 10 realizations (only one realization is depicted).

The ground truth $\boldsymbol {\tau }$ and reconstructions at different count levels for simulation subject 4 are shown in Fig. 2. No other simulation subjects were used for network training.

There are two different input cases (3 M or 30 M counts) and two outputs (300 M or $\boldsymbol {\tau }$), resulting in four different combinations. Test metrics are all calculated against the ground truth $\boldsymbol {\tau }$ (even in the case of 300 M count targets).

**Fig. 3.**
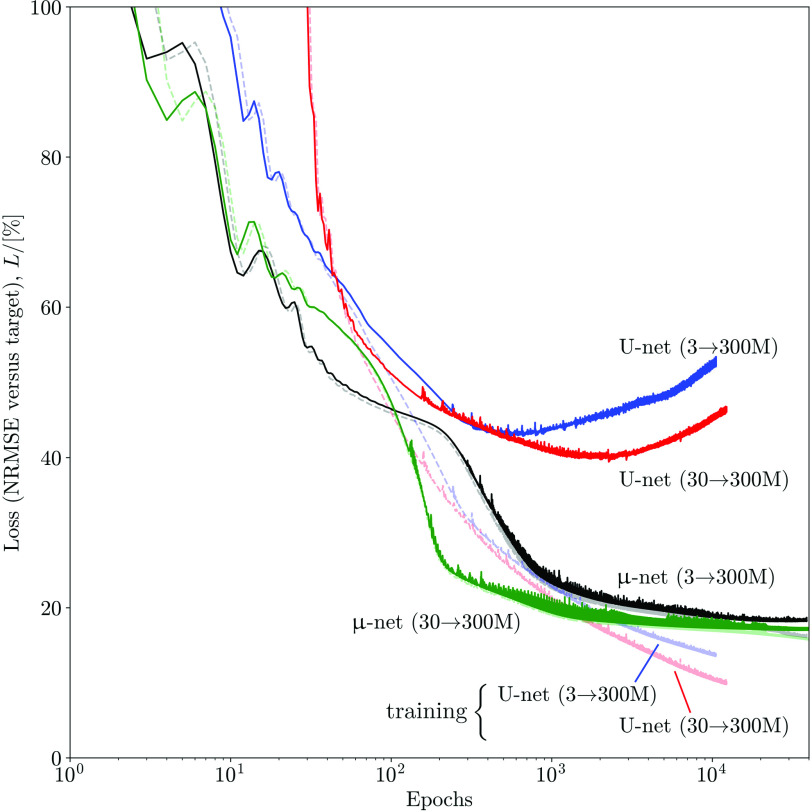
Simulation loss curves for high (300 M) count targets during training on subject 4 (pale dashed lines) and validation on subject 5 (solid lines).

Four $\mu $-nets are trained separately (one for each input-output combination). Four U-nets are also trained for comparison. The loss curves for the 300 M output cases are shown in Fig. 3.

Note that for each network, the input channels are as described in Section II-B2 (four channels: low count reconstructions with and without RM; T1-weighted MR, and T1-guided NLM filtering of the RM reconstruction).

Note that the U-net eventually achieves much lower training loss (due to its increased learning capacity) compared to the $\mu $-net. However, the U-net easily overfits after around 50 epochs, where validation and training losses start to diverge. By comparison, when using the same data, the $\mu $-net validation curves lie almost perfectly on top of the corresponding training curves. This demonstrates a far superior robustness against overfitting with limited amounts of training data.

**Fig. 4.**
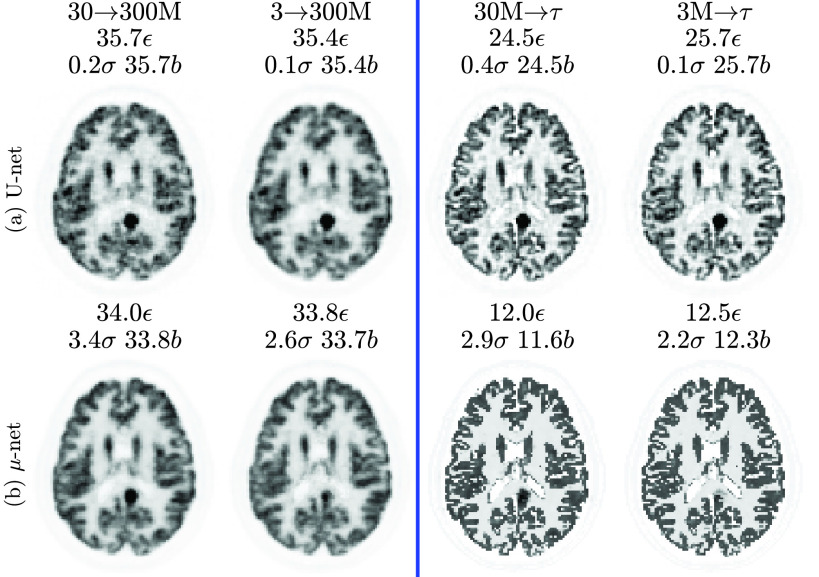
Simulation **training** data predictions (compare to Fig. 2). Note that the U-net has higher errors (than the $\mu$-net) due to early termination of training (at minimum validation loss).

The final training outputs (predictions based on training data from Fig. 2) for all four input-output cases are shown in Fig. 4.

While both $\mu $-nets and U-nets are capable of matching a 300 M count target, it is interesting to note that the $\mu $-nets have half the NRMSE for a ground truth target. This is because of the early termination of training (at minimum validation loss). Fig. 3 shows that for the $\mu $-nets, this corresponds to a similarly stable and low training loss. However, for the U-nets, training loss is still relatively high and decreasing when minimum validation loss is achieved. Training the U-nets further produces much lower training losses at the cost of higher validation losses (and thus reduced generalizability and robustness to unseen test data).

For a fair comparison to the proposed method, the smoothing kernel width (mm FWHM) and NLM parameter ($\Omega $) are found by numerically minimizing NRMSE versus the relevant target $\boldsymbol {T}$ over the training data set (subject 4).

**Fig. 5.**
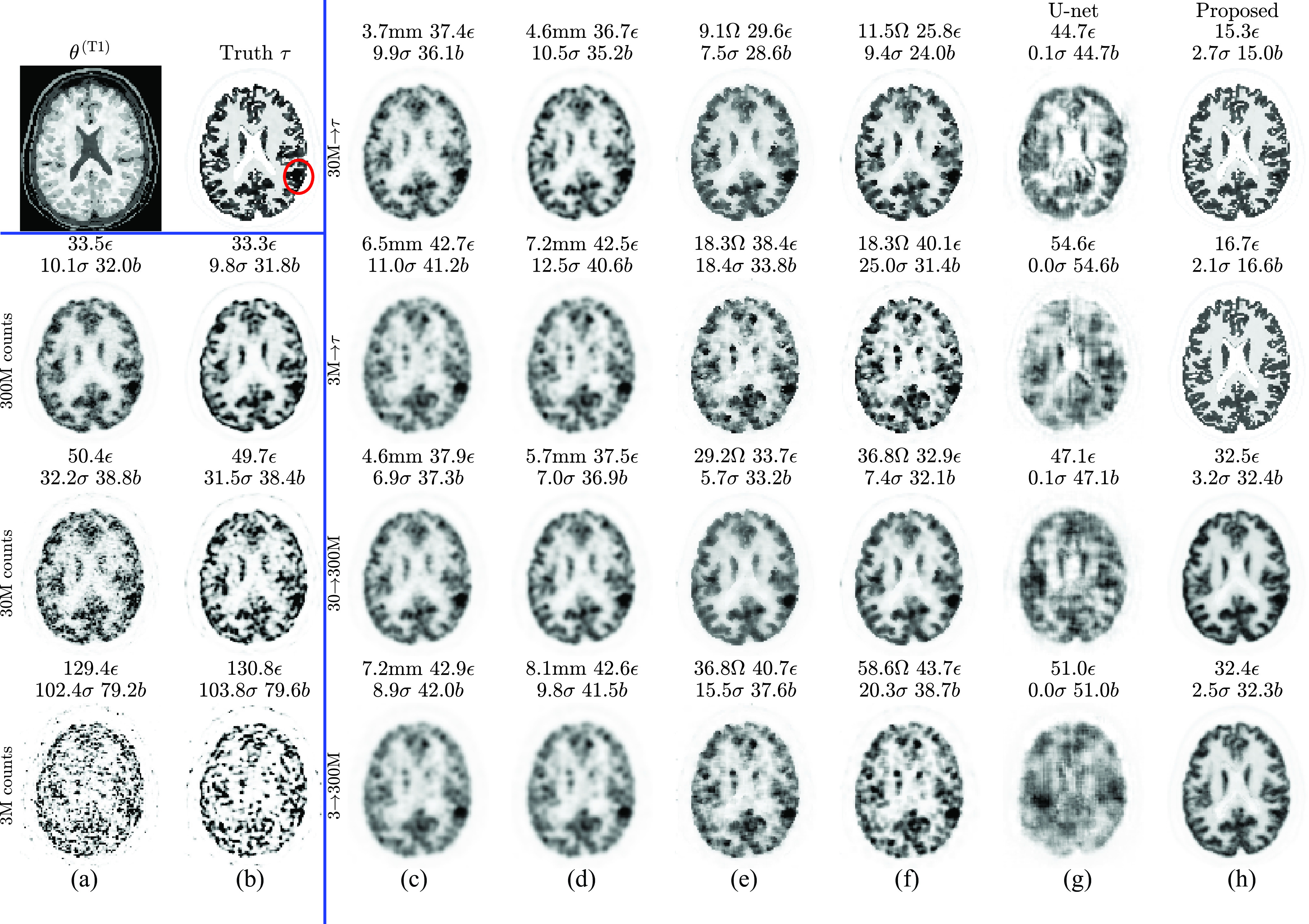
Simulation **test** data: cropped central slices from one set of MLEM reconstructions of subject six at different count levels without (a) and with (b) RM. For comparison (c)–(f) and proposed (h) methods, optimization is performed to minimize NRMSE between the training input and target. This is given by the row titles, which are labeled according to “input $\rightarrow$ optimization target” (see Fig. 2 for corresponding training data images). NRMSE $\epsilon$ and bias $b$ metrics are calculated versus the known ground truth $\boldsymbol\tau$. Standard deviation $\sigma$ is across ten realizations. Optimal values are given in panel titles for smoothing FWHM (mm) and NLM parameter ($\Omega$).

Predictions are made based on test data (ten realizations each for 18 subjects). Results for subject 6 are shown in Fig. 5. The best of the competitive methods is NLM performed on RM, except for the mapping of $\mathrm {30~M}\rightarrow \mathrm {300~M}$ counts, where PS on RM produces a lower NRMSE. In all cases, the proposed method has a lower NRMSE and visually fewer artefacts.

**Fig. 6.**
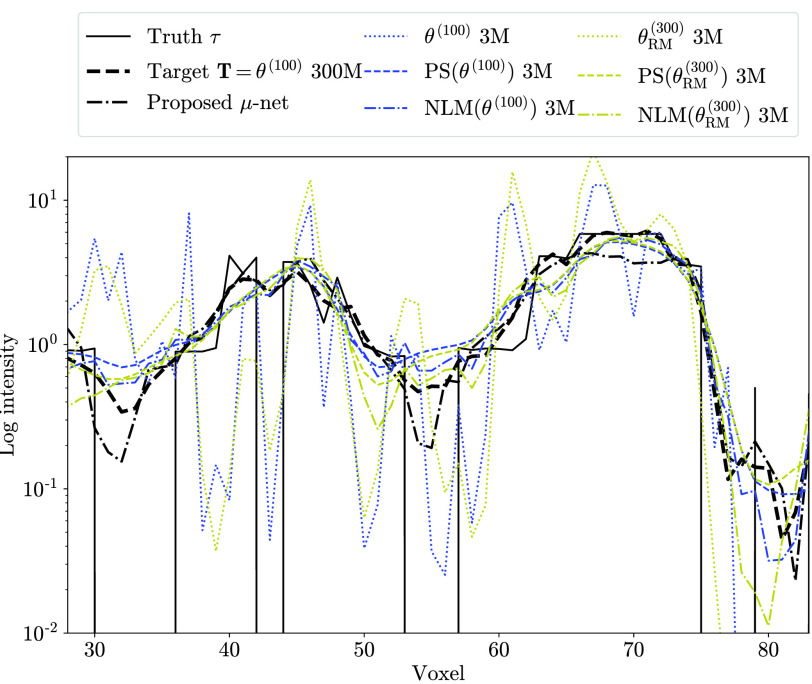
Test data profiles (horizontal line through the lesion circled in Fig. 5 $\boldsymbol{\tau}$) for $\mathrm{3~M}\to\mathrm{300~M}$ counts mapping.

Profiles including the lesion in Fig. 5 are shown in Fig. 6. Note that the $\mu $-net simultaneously suppresses noise, partial volume, and ringing effects to match the standard count reconstruction.

**Fig. 7.**
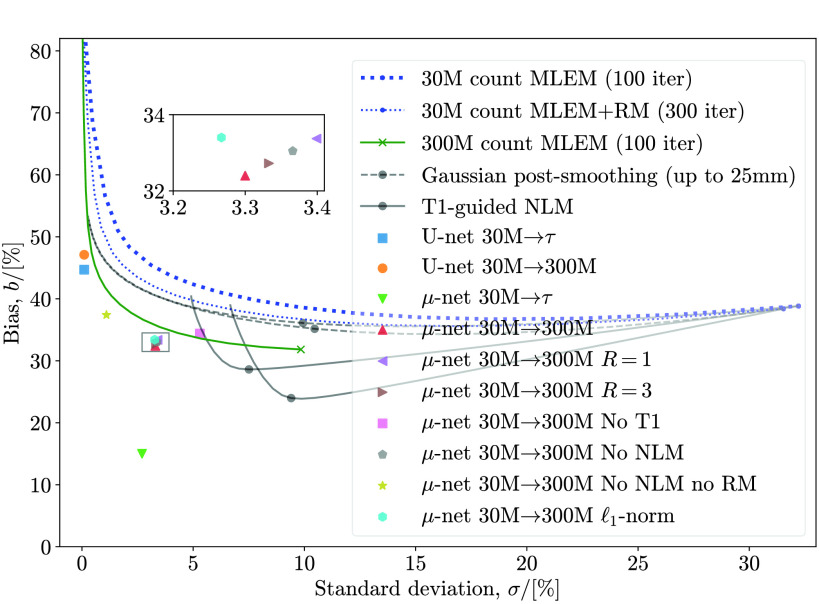
Test bias versus standard deviation. Distance from the origin corresponds to NRMSE (note that the axes have different scales). Standard (300 M) and low (30 M) count curves show the tradeoff with increasing MLEM iterations (endpoints marked with crosses). Gaussian PS of increasing FWHM and NLM filtering with increasing $\Omega$ are also depicted with optimal values circled. The proposed network’s prediction based on low count inputs has comparable bias and much lower standard deviation compared to the target standard count reconstruction. Unless specified otherwise, $R=2$ realizations of one patient were used to train each network.

Fig. 7 shows bias versus standard deviation curves with increasing MLEM iterations for 30 M count inputs. The effects of Gaussian PS of the endpoints of MLEM reconstructions are also shown for FWHM increasing in steps of 0.1 mm. NLM filtering is also applied for $\Omega \in [10^{-5}, 10^{5}]$ in logarithmic steps (increments on the exponent) of 0.01. Optimal (closest to the origin, identical to minimal NRMSE) values are clearly marked. The network’s output (based on low count MLEM endpoints) is comparable to the target MLEM endpoint.

The effects of different network input channels are also investigated. Various inputs are replaced with zeros and in each case the network was retrained. Note that removing inputs altogether instead would change the network architecture. Zeroing inputs has a detrimental effect on test error in all cases. Excluding T1 information (also excluding T1-guided NLM; purely supplying MLEM and MLEM+RM) is slightly better than not, including NLM and MLEM+RM (purely supplying MLEM and T1). This is interesting as it implies that (for the given noise level) RM is more important for quality improvement than T1 information. Ideally, the networks should be retrained several times in order to produce confidence intervals to verify this.

An $\ell _{1}$-norm may be used instead of $\ell _{2}$ (9) “to encourage less blurring” [52]. While both would be susceptible to noise, $\ell _{1}$ may be less so. We have thus also included results for an otherwise identical $\mu $-net trained with an $\ell _{1}$ loss function for comparison. As expected, this results in a slightly higher NRMSE (minimizing $\ell _{2}$ is identical to minimizing NRMSE, unlike $\ell _{1}$).

Furthermore, it is interesting to note that retraining the network with more ($R=3$) realizations evidently has negligible improvement, while using fewer ($R=1$) has very little detriment.

Note that a network trained to match the ground truth $\boldsymbol {\tau }$ (also shown) has built-in information about reconstruction bias which neither PS nor NLM alone could compensate for.

**Fig. 8.**
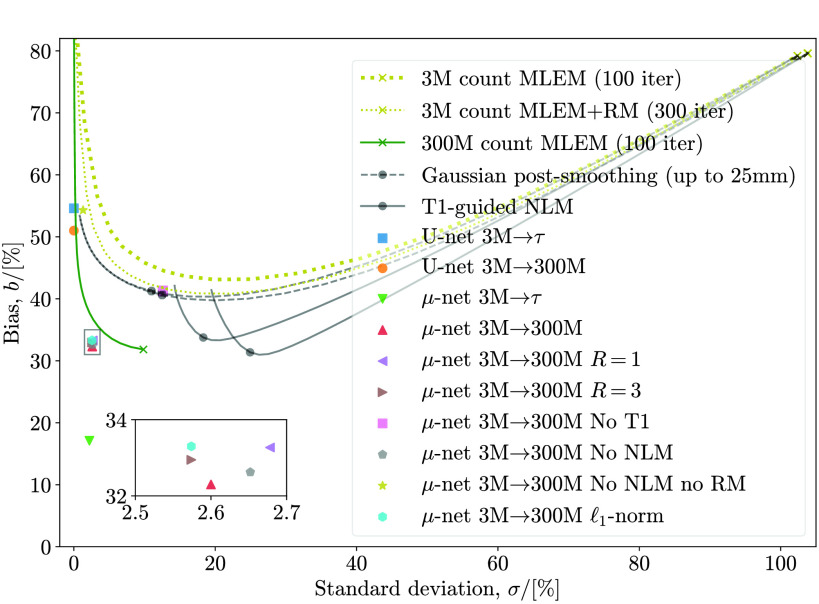
Test bias versus standard deviation for very low (3 M) counts, similar to Fig. 7.

A similar graph for 3 M counts is shown in Fig. 8. This makes it clearer that omitting RM information harms network performance more than omitting T1 information does. There is also a slight improvement as training realizations $R$ increase from 1 to 2, and a negligible improvement from 2 to 3.

**Fig. 9.**
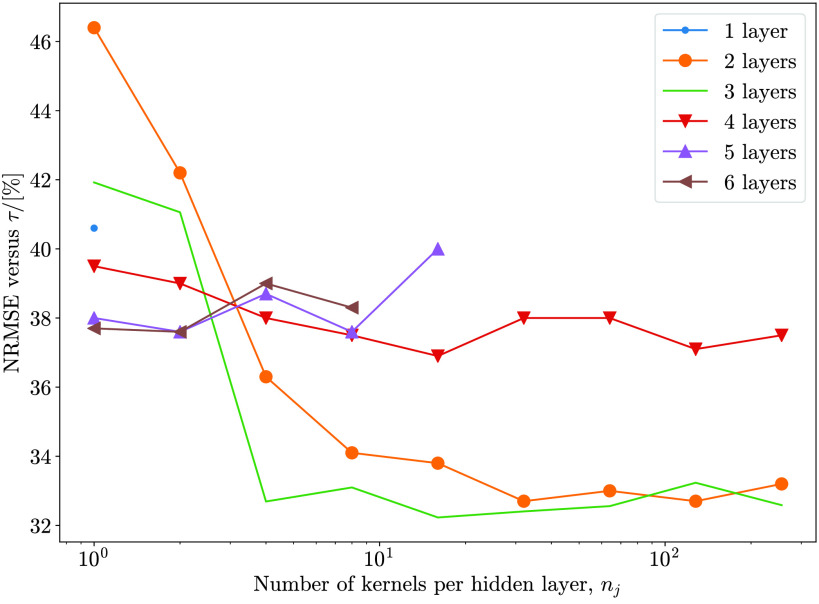
Effect of varying number of layers $J$ and number of kernels per layer $n_{j}$ on **test** NRMSE (for $\mathrm{3~M}\to\mathrm{300~M}$ counts mapping, calculated versus truth $\boldsymbol{\tau}$). For each choice of layers $J$, the number of kernels $n_{j}$ is set to 1 for all hidden layers. The number of kernels per layer $n_{j}$ is then increased from 1 up to 256 in powers of 2 to produce the curves above. Due to memory constraints, it was only possible to reach up to $n_{j}=16$ and 8 kernels per layer for $J=5$ and 6 layers, respectively.

Note that several different $\mu $-networks were trained with various numbers of layers $J$ and choices of kernel numbers $n_{j}$ per layer in order to find an optimal combination. $n_{j}$ were always set to be the same for all hidden [$j\in [1,J)$] layers, and increased from 1 to 256 in powers of 2. Note that the final $n_{J}$ can only be 1 due to requiring only one output channel. An investigation of different architectures showed that $n_{j}=32$ kernels were sufficient in all cases. Fig. 9 shows NRMSE for the case of 3 M to 300 M counts mapping. Error increases slightly for larger $n$. As discussed in Section II-B2, it is possible that this is due to increased optimization difficulty rather than overfitting.

### B. Real Data

**Fig. 10.**
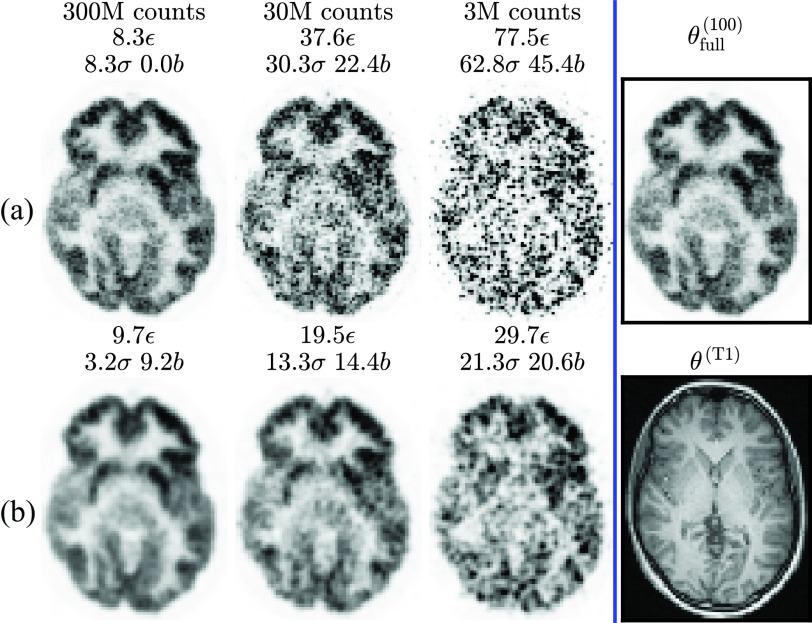
Real patient training data: cropped central slices from MLEM reconstructions of patient 1 following the same layout as Fig. 2. NRMSE $\epsilon$ and bias $b$ are calculated against the full count reconstruction $\boldsymbol{\theta}^{(100)}_{\text{full}}$ (black rectangle), including for the bootstrap sampled $\mathrm{300~M}$ (standard) count target $\boldsymbol T$. Standard deviation $\sigma$ can be estimated since ten realizations were generated for each count level.

Reconstructions for training (patient 1)—similar to the simulations in Fig. 2—are shown in Fig. 10. Standard deviation $\sigma $ can be calculated across multiple realizations by resampling the raw data as mentioned in Section II-A2.

Apart from being based on real PET data acquisitions, a big difference between simulations and real data is the nature of the MR information. The real T1 images are lower resolution, contain artefacts, have different noise properties, and are not perfectly registered.

**Fig. 11.**
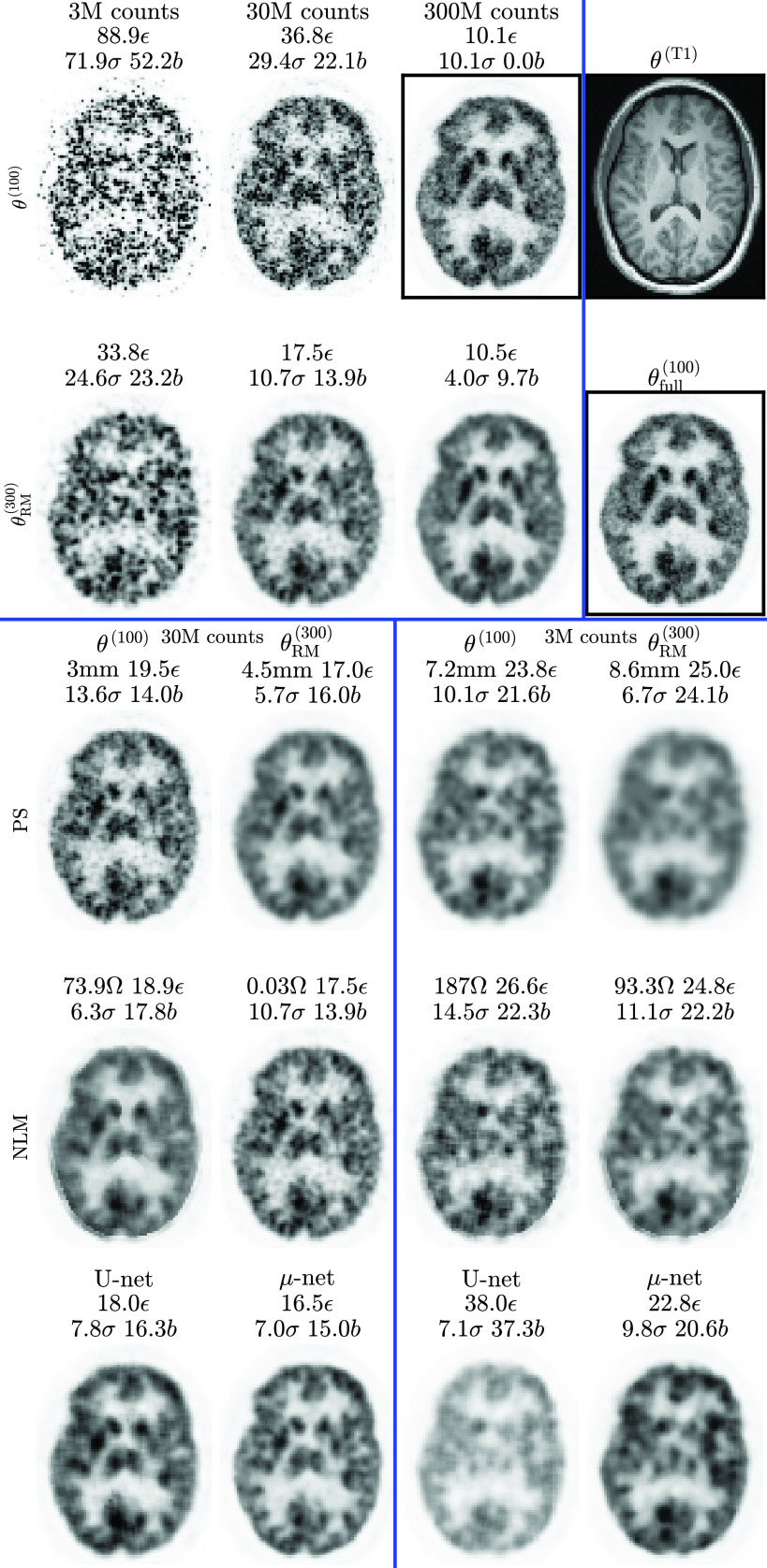
Real patient test results: cropped central slices from MLEM reconstructions of patient 2. There are two images in black boxes: the 300 M reconstruction depicted is a target $\boldsymbol T$ for comparison, while the full count reconstruction $\boldsymbol{\theta}^{(100)}_{\text{full}}$ (without bootstrap sampling) was used as a reference to calculate bias and standard deviation (including for the target $\boldsymbol T$).

Test data and the corresponding $\mu $-net prediction are shown in Fig. 11. Note that since the ground truth is unknown, metrics are calculated with reference to the full count reconstruction $\boldsymbol {\theta }^{(100)}_{\text {full}}$.

## IV. Conclusion

The simulations results clearly show that application of a $\mu $-net always produces lower NRMSE than PS or NLM filtering (see Figs. 7 and 8). The micro-network predictions in Fig. 5(h) also show much less noise—a reduction in standard deviation $\sigma $ by a factor of up to 3 compared to rivals (c)–(f)—and lower bias. The exception is the case of mapping $\mathrm {30~M}\rightarrow \mathrm {300~M}$, where a slightly higher $\sigma $ than NLM is compensated for by the lower bias to still produce a lower overall NRMSE (visible in Fig. 7). This reduction is achieved without sacrificing image resolution.

Future work will need to consider the impact of mismatched noise levels (testing on different noise levels than used for training), as well as using one architecture to compensate for noise and artefacts at different noise levels and at different iterations of MLEM (rather then retraining a network for each case). Increasing the number of training data sets will also produce a more robust network with even better resolution recovery and artefact suppression properties. It would also be interesting to investigate why simply providing more low count reconstructions of the same patient during the training phase (increasing $R$) does not seem to significantly increase network robustness to noise. Generative adversarial networks (GANs), which can be used to augment data sets [53], have been recently applied to low dose PET [52], [54]. It would be particularly interesting in future work to combine the methods proposed here in a GAN framework. The network could also easily be extended to include joint modality (synergistic) post-processing, such as PET-guided undersampled MR reconstruction, or even modality generation such as PET prediction based on MR.
